# miR-149 Inhibits Non-Small-Cell Lung Cancer Cells EMT by Targeting FOXM1

**DOI:** 10.1155/2013/506731

**Published:** 2013-05-16

**Authors:** Yang Ke, Weiyong Zhao, Jie Xiong, Rubo Cao

**Affiliations:** ^1^Cancer Center, Union Hospital, Tongji Medical College, Huazhong University of Science and Technology, Wuhan 430022, China; ^2^Department of Oncology, The Second Affiliated Hospital of Nanjing Medical University, Nanjing 210011, China

## Abstract

MicroRNAs (miRNAs) have been implied to play crucial roles for epithelial-to-mesenchymal transition (EMT) of non-small-cell lung cancer cells (NSCLC cells). Here we found that the expression of miR-149, downregulated in lung cancer, was inversely correlated with invasive capability and the EMT phenotype of NSCLC cells. miR-149 inhibited EMT in NSCLC cells. Furthermore, we demonstrated that miR-149 directly targeted Forkhead box M1 (FOXM1), and FOXM1 was involved in the EMT induced by TGF-**β**1 in A549 cells. Overexpression of FOXM1 restored EMT process inhibited by miR-149. Our work suggested that miR-149 might be an EMT suppressor in NSCLC cells.

## 1. Introduction

Lung cancer was the most frequently occurring malignant cancer, and the leading death cause of lung cancer was metastasis. Tumor metastasis involved several steps including separation, migration, invasion, and formation of a new tumor nodule. Epithelial-to-mesenchymal transition (EMT) played key role in the process of cancer metastasis [1]. EMT not only changed cell morphology but also induced cells to acquire essential new functions like migration and invasion. EMT was characterized by different regulations of epithelial and mesenchymal genes. The increase of mesenchymal markers vimentin and N-cadherin and the loss of epithelial marker E-cadherin were associated with EMT [2, 3]. Transforming growth factor-*β*1 (TGF-*β*1) had been suggested to be an important inducer of EMT in various cancers [4].

MicroRNAs (miRNAs) were a class of small noncoding RNAs, ~18–25 nucleotides, which regulated gene expression posttranscriptionally via inhibiting translation or inducing target mRNA degradation. Dysregulation of miRNAs occurred in a variety of cancers, and several miRNAs acted as essential modulators for EMT in non-small-cell lung cancer (NSCLC). Previous studies reported a downregulation of miR-149 in NSCLC, and miR-149 functioned as a tumor suppressor in human glioma and gastric cancer [5–7]. However, the role of miR-149 in the EMT of NSCLC cells remained poorly understood.

In the present study, we found that miR-149 was significantly downregulated and the expression of miR-149 was inversely correlated with the invasive capability and EMT phenotype of NSCLC cells. Ectopic expression of miR-149 inhibited EMT in NSCLC cells. Furthermore, we found that miR-149 directly targeted FOXM1, a potential metastasis promoter.

## 2. Materials and Methods

### 2.1. Cell Lines

Four NSCLC cell lines including A549, Calu3, Calu1, and H1299 were obtained from ATCC. These cells were grown in RPMI 1640 media (Whitaker Biomedical Products, Whitaker, CA, USA) containing 10% fetal bovine serum (FBS), 100 *μ*g/mL streptomycin/penicillin. To induce EMT, cells were treated with 5 ng/mL TGF-*β*1.

### 2.2. Plasmids

Wildtype 3′ untranslated region (3′UTR) of FOXM1 (FOXM1-WT-3′UTR) or the mutant (FOXM1-Mut-3′UTR) containing potential miR-149 target sites were amplified and subcloned into psiCHECK2 vector (Promega, Fitchburg, WI, USA). miR-149 and the vector were obtained from GeneCopoeia (Rockville, MI, USA). Anti-miR-149 and anticontrol were purchased from RiboBio (Guangzhou, China). FOXM1-shRNA and the control shRNA were purchased from GeneChem (Shanghai, China). pcDNA3-FOXM1 was gifted by Dr. Sarkar at Wayne State University.

### 2.3. RNA Extraction and Quantitative PCR

Total RNA was extracted using Trizol (Invitrogen, Carlsbad, CA, USA) according to the manufacturer's instruction. 1 *μ*g RNA was traversely transcripted, and quantitative PCR was performed using ABI 7500 Sequence Detection System (Life Technologies, NY, USA). Expression level of miRNAs was normalized by U6.

### 2.4. Luciferase Reporter Assays

H1299 cells were co-transfected with 0.5 *μ*g of the reporter vector (psiCHECK-2, psiCHECK-2- FOXM1-WT-3′UTR or psiCHECK-2-FOXM1-WT-3′UTR) and 1 *μ*g of miR-149 expression plasmid or the control vector. Fourty-eight hours after transfection, cells were harvested and luciferase activity was detected using a dual-luciferase reporter assay system (Promega, Fitchburg, WI, USA).

### 2.5. Invasion Assays

To examine the effect of miR-149 on the invasion of NSCLC cells, we used a previously described assay employing reconstituted basement membrane matrigel (BD, San Jose, Biosciences, MA, USA) as the substrate for invasion. Briefly, NSCLC cells were transfected with miR-149 and control, or anti-miR-149 and anticontrol and incubated for 48 h at 37°C and then transferred onto the upper invasion chambers (8 *μ*m pore size, 24-well insert) in a serum-free growth medium. Complete growth medium containing 10% FBS was added to the lower chamber as a chemoattractant. Following 24 h of incubation at 37°C, cells on the surface of upper chambers (noninvading cells) were removed by cotton swabs. Cells that invaded the matrigel to the lower membrane were stained with 0.1% crystal violet. Invaded cells were counted under light microscope (Olympus, Tokyo, Japan).

### 2.6. Western Blots

Cells were collected and lysed in RIPA (Beytime, Shanghai, China). Equal amounts of proteins from each group were separated by 10% SDS-PAGE, tank-based transferred to nitrocellulose membrane, and blocked for 0.5 h by TBS containing 3% nonfat milk. The membrane was incubated with the specific primary antibody overnight at 4°C followed by horseradish-peroxidase-linked secondary antibodies and visualized by ECL (Pierce, Rockford, IL, USA). Primary and secondary antibodies were purchased from tech (Wuhan, China).

### 2.7. Immunofluorescence

A549 cells, cultured on cover slip, were transfected with miR-149, control or anti-miR-149, anticontrol, respectively, for 48 h. Cells were washed, fixed in 4% paraformaldehyde for 15 min, and then rinsed in PBS three times for 5 min, blocked with 5% BSA in PBS for 30 min at 25°C. Primary antibody for vimentin was added and incubated for 1 h at 37°C and then detected using FITC-conjugated secondary antibody. Hoechst was used to stain the nuclear. The fluorescent images were obtained under a fluorescence microscope.

### 2.8. Statistical Analysis

SPSS 12.0 software was used for statistical analysis. Results are presented as mean ± SD from three independent experiments. Differences between groups were analyzed by the two-tailed Student's *t*-test. *P* values <0.05 were accepted as statistically significant.

## 3. Results

### 3.1. miR-149 Inversely Correlated with Invasive Capability and EMT Phenotype of NSCLC Cells

**Figure 1:**
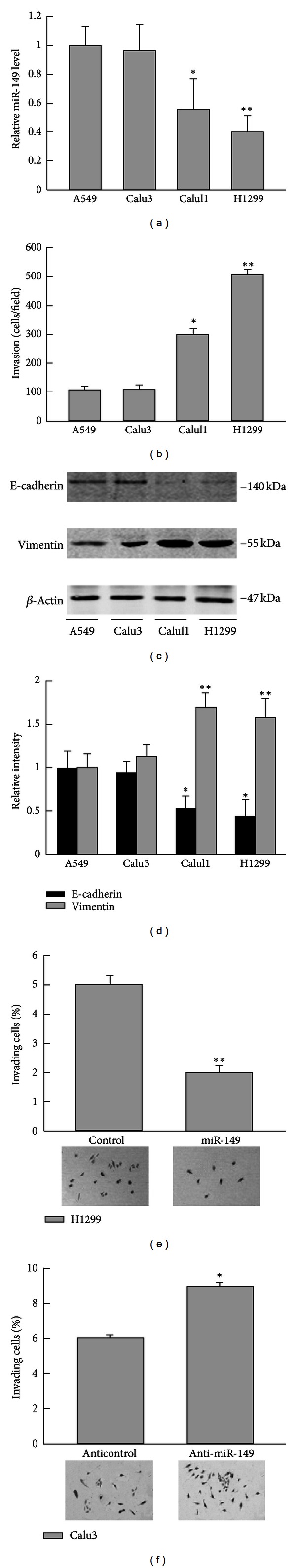
miR-149 inversely correlated with invasive capability and EMT phenotype of NSCLC cells. (a) miR-149 levels in different NSCLC cell lines were measured by quantitative PCR. (b) Invasive capability of NSCLC cells was determined by invasion assay. (c) Western blot analysis of E-cadherin and vimentin in different NSCLC cell lines. (d) Relative intensity of blots in (c). (e) Transfection of miR-149 decreased the invasion of H1299 and increased the invasion of Calu3 cells (f). Data were from three independent experiments. *Compared with A549 or the control group, *P* < 0.05, ^∗ ∗^compared with A549 or the control group, *P* < 0.01.

To investigate the effect of miR-149 on the invasive capability and EMT phenotype in NSCLC cells, A549, Calu3, Calu1, and H1299 four different NSCLC cell lines were used. We found that the level of miR-149 was inversely correlated with invasive potential (Figures 1(a) and 1(b)). Consistent with the invasive capability, higher levels of E-cadherin were expressed in low invasive cells, while highly invasive cell lines expressed higher levels of vimentin (Figures 1(b), 1(c), and 1(d)). 

To further study the role of miR-149 in inhibiting invasion, we transfected H1299 (high invasive, low miR-149) cells with miR-149 and performed matrigel invasion assays. Results showed that miR-149 efficiently inhibits the invasion (Figure 1(e)). On the contrast, Calu3 (low invasive, high miR-149) cells treated with anti-miR-149, inhibitor of miR-149, showed significantly increased invasion (Figure 1(f)). These results implied that downregulation of miR-149 might contribute to the invasive phenotype in NSCLC cells.

### 3.2. miR-149 Inhibited EMT in NSCLC Cells

**Figure 2:**
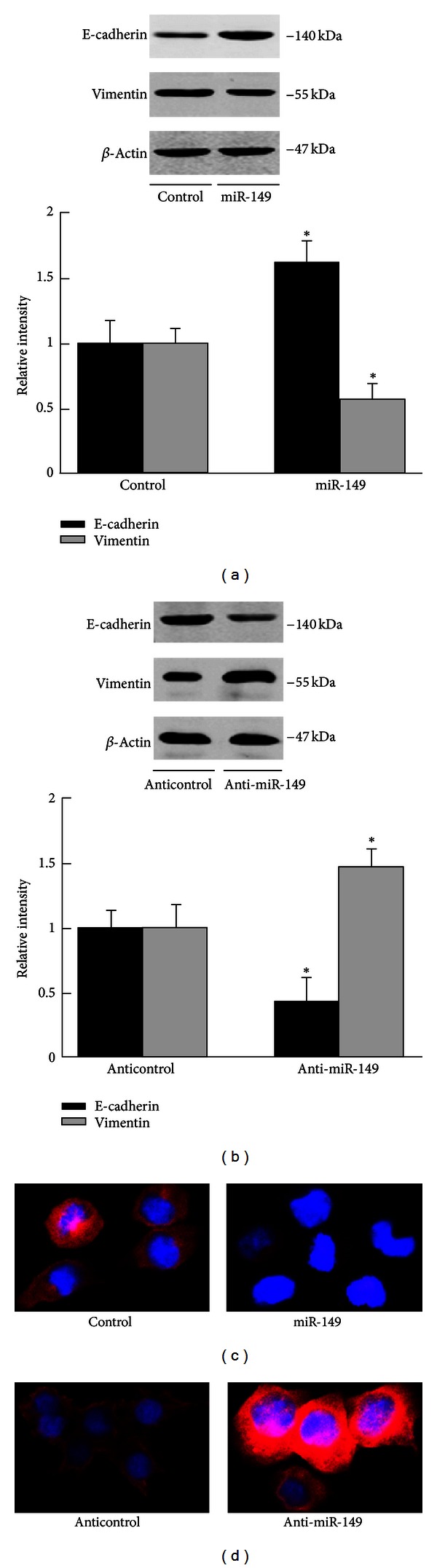
miR-149 inhibited EMT in NSCLC cells. (a) miR-149 increased E-cadherin expression and decreased vimentin expression in H1299 cells. (b) Anti-miR-149 decreased E-cadherin expression and increased vimentin expression in A549 cells. Data were from three independent experiments. (c) Immunofluorescence of vimentin in A549 transfected with miR-149 or the control. (d) Immunofluorescence of vimentin in A549 transfected with anti-miR-149 or the control. *Compared with the control group, *P* < 0.05.

Ectopic transfection of miR-149 in H1299 cells decreased vimentin expression and increased E-cadherin expression (Figure 2(a)). The A549 (low invasive, high miR-149) cells treated with anti-miR-149 showed an increase in vimentin expression and a decrease in E-cadherin expression (Figure 2(b)). Immunofluorescence of vimentin was consistent with the western blot results (Figures 2(c) and 2(d)). These data were consistent with the effect of miR-149 on the invasion phenotype in NSCLC cells.

### 3.3. miR-149 Directly Targeted FOXM1

**Figure 3:**
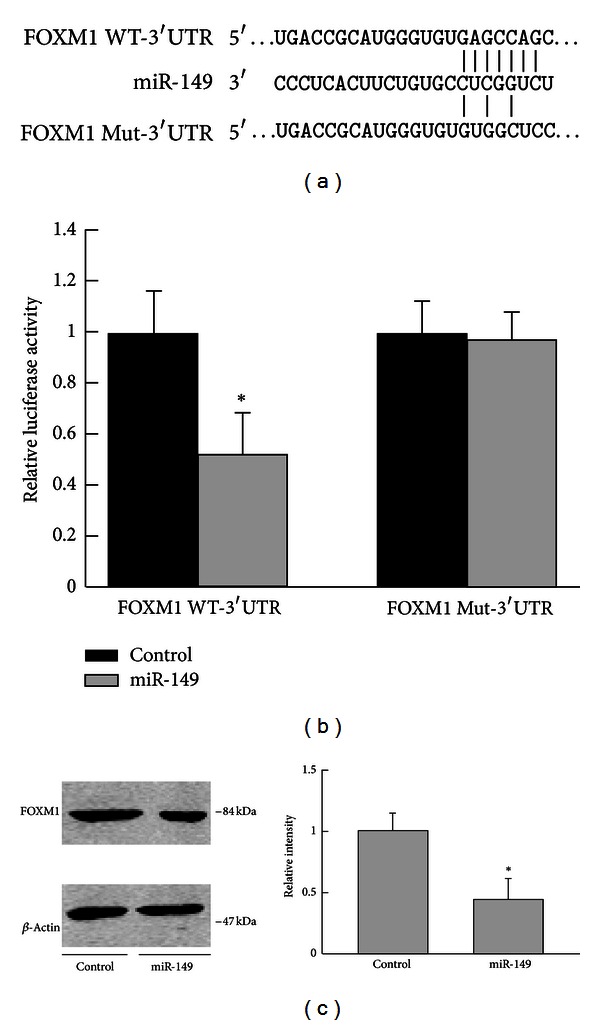
miR-149 directly targeted FOXM1. (a) The predicted miR-149 target site in the 3′UTR of FOXM1 mRNA and its mutated version. (b) Luciferase activity assays in H1299 cells showed that miR-149 inhibited the expression of FOXM1. (c) miR-149 decreased FOXM1 expression in H1299 cells. Data were from three independent experiments. *Compared with the control group, *P* < 0.05.

miRNA target analysis tools PicTar and TargetScan 6.2 were used to explore potential target of miR-149. FOXM1 was predicted to be a target of miR-149 (Figure 3(a)). To validate targeting of FOXM1 by miR-149, we performed luciferase activity assay. The wildtype FOXM1 3′UTR luciferase reporter plasmid (WT-3′UTR) or the mutated (Mut-3′UTR) was cotransfected with miR-149 overexpression plasmid or the control vector into H1299 cells. The cotransfection of miR-149 with WT-3′UTR in H1299 cells showed significant decreased luciferase activity compared with the control vector group, while Mut-3′UTR luciferase activity was not changed (Figure 3(b)). Overexpression of miR-149 in H1299 cells significantly decreased the protein level of FOXM1 (Figure 3(c)). These results suggested that miR-149 inhibits FOXM1 expression.

### 3.4. FOXM1 Was Involved in EMT of A549 Cells Induced by TGF-*β*1

**Figure 4:**
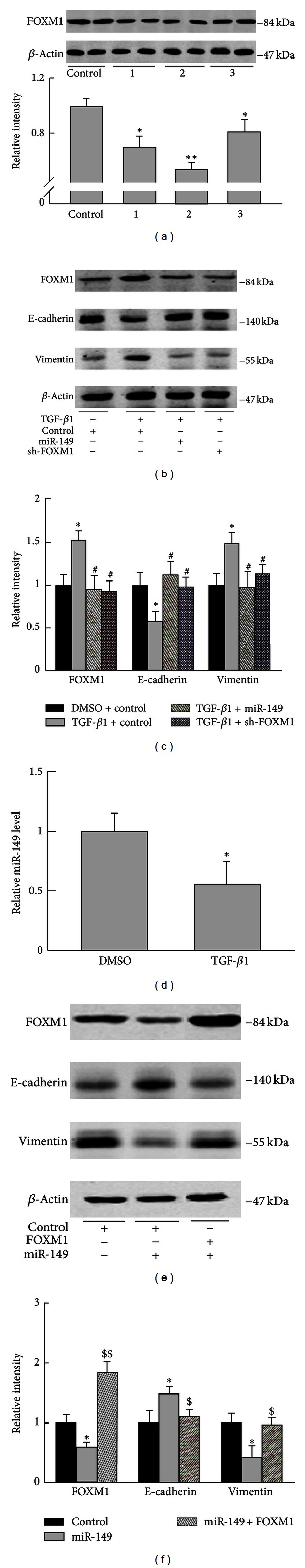
FOXM1 was involved in EMT of A549 cells induced by TGF-*β*1. (a) A549 cells were transfected with sh-control, sh-FOXM1 1~3, respectively, for 48 h and harvested for western blot. sh-FOXM1 2 showed the most inhibitory effect and was selected for the following experiments. (b) A549 cells were transfected with control, sh-FOXM1, or miR-149, respectively, and incubated for 24 h, then TGF-*β*1 (5 ng/mL) was added to the medium, and cells were harvested 48 h later. Western blot examined the protein levels of FOXM1, E-cadherin, and vimentin. (c) Relative intensity of blots in (b) was analyzed. (d) A549 cells were treated with DMSO or TGF-*β*1 (5 ng/mL) for 24 h and collected for microRNA extraction. miR-134 expression was examined. (e) A549 cells were transfected with control, miR-149, or cotransfection of miR-149 and FOXM1 for 48 h. Cells were harvested for western blot analysis. (f) Relative intensity of blots in (e) was analyzed. Data were from three independent experiments. *P < 0.05, compared with control group; ^#^ *P* < 0.05, ^##^ *P* < 0.01, compared with TGF-*β*1 group; ^$^ *P* < 0.05, ^$$^ *P* < 0.01 compared with miR-149 group.

To further study the role of FOXM1 in EMT process, we treated A549 cells with 5 ng/mL TGF-*β*1. miR-149 overexpression reversed the expression of vimentin and E-cadherin induced by 5 ng/mL TGF-*β*1 treatment (Figure 4(b)). We used sh-FOXM1 to suppress FOXM1 expression. As shown in Figure 4(a), three constructs were generated and the effect was examined by western blot. Similar effect sh-FOXM1 was observed in TGF-*β*1 treated cells, suggesting that sh-FOXM1 could mimic the effect of miR-149. In addition, TGF-*β*1 treatment dramatically increased FOXM1 expression, while decreasing miR-149 expression (Figures 4(b), 4(c), and 4(d)). Ectopic expression of FOXM1 could restore EMT inhibited by miR-149 (Figures 4(e) and 4(f)). These data implied that miR-149 might inhibit EMT by suppressing FOXM1 expression in NSCLC cells.

## 4. Discussion

NSCLCs accounted for 80% of lung cancer, the main cause of cancer mortality worldwide [8]. The prognosis of NSCLC patients remained low despite great advances had occurred in early diagnosis and chemo/targeted therapies [9]. NSCLC tumor metastasis was a complex, multistep process induced by genetic or epigenetic changes [10]. EMT was an essential event in tumor progression and metastasis, characterized by changes of epithelial and mesenchymal maker gene expression, as well as morphology changes. The regulatory networks that control EMT have not been well illustrated. 

Emerging evidences implied that miRNAs play crucial roles in the regulation of carcinogenesis and EMT of cancer cells [11]. Altered miRNA levels have been found in the initiation or development process of cancers. Expression profiles of miRNAs have been studied by several groups [12, 13]. Many miRNAs have been identified to promote EMT. All members of the miR-17–29 cluster located in 13q31.3 could accelerate tumor metastasis by cooperating with c-Myc [14]. miR-23a suppressed E-cadherin expression and stimulated EMT under the stimulation of TGF-*β*1 [15]. Liu et al. demonstrated that miR-31 promoted EMT by repressing the expression of tumour suppressor large tumor suppressor 2 [16]. Conversely, a variety of miRNAs have been suggested to be suppressors of metastasis via inhibiting EMT process. Particularly, the miR-200 family members have been demonstrated to resist EMT and promote epithelial differentiation [17]. Li et al. found that miR-134 significantly inhibited EMT of NSCLC cells by targeting FOXM1 [18]. 

Recent studies have implicated the essential role of miR-149 in various diseases, including the progression of various types of malignant tumors. However, the results were controversial [19, 20]. miR-149 expression was decreased in some tumors, including NSCLC, and functioned as a tumor suppressor by inhibiting oncogenes expression. For example, in glioblastoma miR-149 was downregulated and it inhibited the proliferation and invasion of glioma cells by blocking AKT1 signaling [7]. Loss of miR-149 resulted in the gain of oncogenes expression and correlated with tumor grade in astrocytomas and renal cell carcinoma [21]. In contrast, oncogenic role of miR-149 was also observed in several cancers. For instance, in melanoma upregulated miR-149 suppressed GSK3-*α* expression and upregulated Mcl-1 expression, resulting in apoptotic resistance [22]. Elevated miR-149 also played essential role in the progression of nasopharyngeal carcinoma [23]. However, little is known about the role of miR-149 in NSCLC. Our study demonstrated that miR-149 expression was inversely correlated with invasive capability and EMT phenotype of NSCLC cells. miR-149 negatively regulated EMT by suppressing FOXM1 expression.

FOXM1 belonged to a member of transcriptional factors characterized by a DNA-binding domain known as the Forkhead box. FOXM1 was expressed in numerous tumor cell lines and regulated the expression of genes involved in cell cycle [24]. Increased FOXM1 had been found in several tumors including brain, liver, lung, colon, breast, and pancreas, suggesting a role as protooncogene in human carcinogenesis [25–27]. Moreover, increased level of FOXM1 coincided with metastasis of many cancers, and FOXM1 had been suggested as a predictor of poor prognosis in cancers [27, 28]. Previous studies showed that FOXM1 induced chemotherapy resistance in NSCLC cells [24]. Our study found that overexpression of miR-149 or knockdown of FOXM1 by shRNA suppressed EMT in NSCLC cells, and miR-149 inhibited FOXM1 expression. In addition, we demonstrated that FOXM1 was involved in EMT of A549 cells induced by TGF-*β*1. These data suggested that increased FOXM1 expression partially due to miR-149 downregulation might contribute to NSCLC cells metastasis.

In summary, the results from this work demonstrated that miR-149 might act as a tumor suppressor in NSCLC cells via inhibiting the expression of FOXM1. Therefore, restoration of miR-149 might provide a therapeutic strategy for NSCLC treatment.

## References

[1] Gao D, Vahdat LT, Wong S, Chang JC, Mittal V (2012). Microenvironmental regulation of epithelial-mesenchymal transitions in cancer. *Cancer Research*.

[2] Kim MA, Lee HS, Lee HE, Kim JH, Yang HK, Kim WH (2009). Prognostic importance of epithelial-mesenchymal transition-related protein expression in gastric carcinoma. *Histopathology*.

[3] Nagathihalli NS, Merchant NB (2012). Src-mediated regulation of E-cadherin and EMT in pancreatic cancer. *Frontiers in Bioscience*.

[4] Fuxe J, Karlsson MC (2012). TGF-beta-induced epithelial-mesenchymal transition: a link between cancer and inflammation. *Seminars in Cancer Biology*.

[5] Mallick R, Patnaik S, Yendamuri S (2010). MicroRNAs and lung cancer: biology and applications in diagnosis and prognosis. *Journal of Carcinogenesis*.

[6] Zaninovic V, Zamora T, Tajima K (1990). Origins of T-cell leukaemia virus. *Nature*.

[7] Pan SJ, Zhan SK, Pei BG, Sun QF, Bian LG, Sun BM (2012). MicroRNA-149 inhibits proliferation and invasion of glioma cells via blockade of AKT1 signaling. *International Journal of Immunopathology and Pharmacology*.

[8] Koshiol J, Rotunno M, Consonni D (2009). Chronic obstructive pulmonary disease and altered risk of lung cancer in a population-based case-control study. *PLoS ONE*.

[9] Angulo M, Lecuona E, Sznajder JI (2012). Role of MicroRNAs in lung disease. *Archivos de Bronconeumología*.

[10] Gupta GP, Massagué J (2006). Cancer Metastasis: building a Framework. *Cell*.

[11] Lin PY, Yu SL, Yang PC (2010). MicroRNA in lung cancer. *British Journal of Cancer*.

[12] Solomides CC, Evans BJ, Navenot JM, Vadigepalli R, Peiper SC, Wang ZX (2012). MicroRNA profiling in lung cancer reveals new molecular markers for diagnosis. *Acta Cytologica*.

[13] Zhang YK, Zhu WY, He JY (2012). miRNAs expression profiling to distinguish lung squamous-cell carcinoma from adenocarcinoma subtypes. *Journal of Cancer Research and Clinical Oncology*.

[14] He L, Thomson JM, Hemann MT (2005). A microRNA polycistron as a potential human oncogene. *Nature*.

[15] Cao M, Seike M, Soeno C (2012). MiR-23a regulates TGF-*β*-induced epithelial-mesenchymal transition by targeting E-cadherin in lung cancer cells. *International Journal of Oncology*.

[16] Liu X, Sempere LF, Ouyang H (2010). MicroRNA-31 functions as an oncogenic microRNA in mouse and human lung cancer cells by repressing specific tumor suppressors. *Journal of Clinical Investigation*.

[17] Brabletz S, Brabletz T (2010). The ZEB/miR-200 feedback loop-a motor of cellular plasticity in development and cancer?. *EMBO Reports*.

[18] Li J, Wang Y, Luo J (2012). miR-134 inhibits epithelial to mesenchymal transition by targeting FOXM1 in non-small cell lung cancer cells. *FEBS Letters*.

[19] Zhang J, Liu YF, Gan Y (2012). Lack of association between miR-149 C>T polymorphism and cancer susceptibility: a meta-analysis based on 4,677 cases and 4,830 controls. *Molecular Biology Reports*.

[20] Wang Y, Zheng X, Zhang Z (2012). MicroRNA-149 inhibits proliferation and cell cycle progression through the targeting of ZBTB2 in human gastric cancer. *PLoS ONE*.

[21] Li D, Chen P, Li XY (2011). Grade-specific expression profiles of miRNAs/mRNAs and docking study in human grade I-III astrocytomas. *OMICS*.

[22] Jin L, Hu WL, Jiang CC (2011). MicroRNA-149*, a p53-responsive microRNA, functions as an oncogenic regulator in human melanoma. *Proceedings of the National Academy of Sciences of the United States of America*.

[23] Luo Z, Zhang L, Li Z (2012). An in silico analysis of dynamic changes in microRNA expression profiles in stepwise development of nasopharyngeal carcinoma. *BMC Medical Genomics*.

[24] Wang Y, Wen L, Zhao SH, Ai ZH, Guo JZ, Liu WC (2013). FoxM1 expression is significantly associated with cisplatin-based chemotherapy resistance and poor prognosis in advanced non-small cell lung cancer patients. *Lung Cancer*.

[25] Hui MK, Chan KW, Luk JM (2012). Cytoplasmic Forkhead box M1 (FoxM1) in esophageal squamous cell carcinoma significantly correlates with pathological disease stage. *World Journal of Surgery*.

[26] Millour J, Constantinidou D, Stavropoulou AV (2010). FOXM1 is a transcriptional target of ER*α* and has a critical role in breast cancer endocrine sensitivity and resistance. *Oncogene*.

[27] Koo CY, Muir KW, Lam EW (2012). FOXM1: from cancer initiation to progression and treatment. *Biochimica et Biophysica Acta*.

[28] Priller M, Pöschl J, Abrão L (2011). Expression of FoxM1 is required for the proliferation of medulloblastoma cells and indicates worse survival of patients. *Clinical Cancer Research*.

